# Development of a Measure of Sleep, Circadian Rhythms, and Mood: The SCRAM Questionnaire

**DOI:** 10.3389/fpsyg.2017.02105

**Published:** 2017-12-01

**Authors:** Jamie E. M. Byrne, Ben Bullock, Greg Murray

**Affiliations:** Centre for Mental Health, Swinburne University of Technology, Hawthorn, VIC, Australia

**Keywords:** sleep, circadian phase, mood, depression, measure, questionnaire

## Abstract

Sleep quality, circadian phase, and mood are highly interdependent processes. Remarkably, there is currently no self-report questionnaire that measures all three of these clinically significant functions: The aim of this project was to address this deficit. In Study 1, 720 participants completed a set of potential items was generated from existing questionnaires in each of the three domains and refined to follow a single presentation format. Study 2 used an independent sample (*N* = 498) to interrogate the latent structure. Exploratory factor analysis was used to identify a parsimonious, three-factor latent structure. Following item reduction, the optimal representation of sleep quality, circadian phase, and mood was captured by a questionnaire with three 5-item scales: Depressed Mood, Morningness, and Good Sleep. Confirmatory factor analysis found the three-scale structure provided adequate fit. In both samples, Morningness and Good Sleep were positively associated, and each was negatively associated with the Depressed Mood scale. Further research is now required to quantify the convergent and discriminant validity of its three face-valid and structurally replicated scales. The new sleep, circadian rhythms, and mood (SCRAM) questionnaire is the first instrument to conjointly measure sleep quality, circadian phase, and mood processes, and has significant potential as a clinical tool.

## Introduction

A range of evidence in normative and clinical populations demonstrates that sleep quality, circadian phase, and mood are highly interdependent (Mason and Harvey, 2014; Soehner et al., 2014, 2016). In clinical settings this generates a common problem: When a patient presents with some combination of sleep quality, circadian phase, and mood problems, where should treatment be targeted (Harvey, 2015)? At least in part, this clinical problem arises from the fact that, while well-validated measures of sleep quality, circadian phase, and mood exist, there is no self-report instrument that quantifies function in all three domains. Such an instrument would provide a quantum advance over existing single-construct measures that, while validly capturing the individual construct of interest, pay no attention to independent and overlapping variance arising from the mechanistic interplay between the three processes. The aim of this project was to take the first step toward addressing this problem by developing a novel self-report questionnaire to measure sleep quality, circadian phase, and mood processes.

There are strong reasons to consider the interaction of sleep quality, circadian phase, and mood problems in treatment. Circadian phase refers to the timing of endogenous circadian rhythms relative to 24-h clock time (Czeisler et al., 1989; Kripke et al., 2007). Circadian phase has reliable associations with diurnal preference of activity and rest, or chronotype (Duffy et al., 2001; Mongrain et al., 2004). Chronotype is anchored by extremes of “morningness” and “eveningness.” Morningness being the preference for earlier activity and sleep times, eveningness the preference for later activity and sleep times (Duffy et al., 1999; Baehr et al., 2000). Sleep quality involves a subjective assessment of total sleep duration, sleep latency, wake after sleep onset, and qualitative interpretation of the depth and restfulness of the sleep period (Buysse et al., 1989). Sleep quality and circadian phase are interdependent processes with individuals high on morningness reporting better sleep quality (Wittmann et al., 2006; Barclay et al., 2016). Moreover, a stable circadian phase, i.e., having regular sleep times, has been found to improve sleep quality (Gruber et al., 2011; Harvey et al., 2011), and the circadian system regulates antidepressant and mood stabilizing medication pathways in the brain (McClung, 2013). Data suggests that cognitive behavioral therapy for insomnia can alleviate symptoms of depression (Manber et al., 2008), and in turn Chan et al. (2014) found high levels of eveningness and insomnia were independent risk factors of non-remitting depression. It is clear therefore, that comprehensive consideration of all three processes has the potential to improve case formulation and treatment planning.

Clinical interactions between sleep quality, circadian phase, and mood processes limit current self-report measures. For example, self-report measures of sleep quality (such as the Pittsburgh Sleep Quality Index) may have difficulty in reliably differentiating individuals with depression from primary insomnia (Buysse et al., 1989). Grandner et al. (2006) hypothesized that self-report measures of sleep quality may in part reflect negative cognitive processes and pessimism rather than sleep quality alone. Similarly, individuals with insomnia scored in the mild symptomology bracket of the Beck Depression Inventory-II (Carney et al., 2009). In a study by Abe et al. (2011), 64% of individuals who had a delayed circadian phase (as measured by the Morningness-Eveningness Questionnaire, Horne and Östberg, 1976) had moderate to severe depressive symptoms. Suggesting a bidirectional link, Robillard et al. (2013) observed that a delayed evening sleep phase is common in those with both unipolar and bipolar depression. Finally, shifts in circadian phase (such as those experienced in jetlag and shift-work) leads to sleep quality complaints (Rajaratnam and Arendt, 2001), and poor sleep quality can also impact circadian function (Morin, 2013).

The overarching aim of this project was to develop a single questionnaire to measure the three interconnected processes of sleep quality, circadian phase, and mood. Such a questionnaire has the potential to guide assessment and treatment given the important clinical conundrum that arises when a client presents with a combination of complaints in the three domains. The present project is a critical first step in an ongoing program of research. The aim of Study 1 was to develop a brief three-factor self-report measure that provides maximal measurement separation between the domains of sleep quality, circadian phase and mood. Depressed mood in physical illnesses is common and leads to an increased mortality rate (Katon, 2003); autonomic function (Grimaldi et al., 2016) and obesity (Haus et al., 2016) have both been associated with sleep and circadian disruption. These processes may be linked by fundamental physiological dysregulation (see, for example, Kemp and Quintana, 2013; Beauchaine and Thayer, 2015; Gruber et al., 2015). As part of a preliminary external validation of the measure, we predicted that scale scores from the new instrument would demonstrate intelligible patterns of convergent and divergent validity with single-item measures of sleep, physical, and mental health problems. Study 2 aimed to confirm the measure's theoretical three-factor structure using confirmatory factor analysis.

## Study 1: Method

### Study design and data analysis

Instrument development was guided by the PROMIS standards (Cella et al., 2007). Item generation involved drawing items from existing questionnaires in the three domains, standardizing them to the same response format and making small changes to wording where necessary. Item reduction was achieved by conducting an Exploratory Factor Analysis (EFA) on responses from a predominantly student sample to this draft set of items. Preliminary investigation of external validity was conducted via associations with self-reported physical and mental health outcomes.

### Item generation

To develop a comprehensive list of items measuring the three domains we examined relevant topic areas from which we could generate newer items. Draft items were identified from highly cited self-report measures of sleep quality, circadian phase, and pathological mood (symptoms of depression and also hypo/mania). Nine sleep scales (e.g., PSQI Buysse et al., 1989 and Insomnia Severity Index Bastien et al., 2001); five circadian rhythm questionnaires [e.g., Munich Chronotype Questionnaire (Roenneberg et al., 2003) and Morningness-Eveningness Questionnaire (Horne and Östberg, 1976)], and 20 mood questionnaires [e.g., BDI-II (Beck et al., 1996), Centre for Epidemiological Studies Depression Scale (Radloff, 1977), and The Hospital Anxiety and Depression Scale (Zigmond and Snaith, 1983)] were used. As part of this generative process we created items that had purified wording, with the specific aim of decreasing overlap between the three domains. Items were selected for being face-valid as relatively pure measures of only one of the three processes. Draft items were then reworded to permit a shared response format (below) and/or to improve clarity and brevity. The preliminary item pool contained 170 items, *n* = 58 measuring sleep quality, *n* = 60 measuring circadian phase, and *n* = 52 measuring problems with mood (full list included in the Supplementary Materials).

The given question prompt was: “The following questions ask about your sleep, mood and timing of daily activities. Pick the answer which best describes you **over the past two weeks**.” A 2-week timeframe was deemed appropriate, given the nature of the processes under investigation (e.g., 2 weeks is the minimum timeframe required for DSM-5 diagnosis of Major Depressive Episode (American Psychiatric Association, 2013). A 6-point Likert-type response scale (*Strongly Disagree* to *Strongly Agree*) was selected for the new questionnaire. We chose not to have a middle response option to avoid ambiguity, as middle response options can reflect socially desirable responding, indifference, uncertainty, or non-applicability (Garland, 1991; Nowlis et al., 2002). Six response categories was considered an optimal balance between the higher validity associated with increasing response options and participant response burden (Chang, 1994; Preston and Colman, 2000).

### Exploratory factor analysis

The expected three factor structure was forced onto the item set. For completeness, a range of empirical methods was also used to explore the number of factors to extract (O'Connor, 2000). The scree plot (Tabachnick and Fidell, 2013), minimum average partial test (Velicer, 1976), parallel analysis (Horn, 1965), and Kaiser-Guttman's criteria (Tabachnick and Fidell, 2013) were all examined. Using an orthogonal rotation (varimax), the adequacy of all factor solutions was compared against the following criteria: item communality magnitudes of over 0.40, cross-loadings less than 0.32 and at least five strongly loading (>0.50) items on each factor (Costello and Osborne, 2005).

### Item reduction

To generate the three scale questionnaire, two item reduction steps were applied. First, items were removed if they failed to load with sufficient strength (< 0.32) on any factor, had low communalities (<0.2), or had high cross-loading (>0.3) (Tabachnick and Fidell, 2013). The item content of the final three-scale questionnaire was developed from the remaining 98 items using the following principles: (1) each scale should have the minimum number of items for internal reliability, (2) each scale should contain reverse-coded items, and equal numbers of reverse-coded items were required for each scale, and (3) heterogeneity of meaning across items was sought to cover the breadth of the domain being measured. When items with comparable loadings were semantically similar, brevity and face validity were preferred.

### Preliminary investigation of external correlates

Participants completed standard demographic questions including age and gender, and were asked dichotomous (Yes/No) questions about physical and mental health problems. For example, “Have you ever been diagnosed with a mental disorder”? Affirmative responses were followed by a request for participants to describe the mental disorder in an open response format. Age, gender, physical health problems, history of mental illness, and self-reported difficulties sleeping were investigated as external correlates of the final questionnaire.

### Participants

A predominantly university student sample (18 years or older) was recruited. A total of 890 individuals commenced the questionnaire package online, with 783 (88%) complete responses recorded (see Table 1 for sample characteristics). Participants were largely first-year university students who were participating in a research experience program (which included on-campus and online students). Other recruitment methods included advertisements on social media and through contacts of the researchers.

**Table 1:** Demographic Characteristics for Study 1 and Study 2.

	**Study 1 (*n* = 720)**	**Study 2 (*n* = 462)**
	***n* (%)**	***n* (%)**
Age (years, M ± SD)	33.32 (10.11)	33.82 ± 11.19
Gender (% Women)	593 (82.4)	366 (79.2)
Employment		
Full-time	256 (35.6)	158 (34.2)
Part-time/ Casual	285 (39.6)	173 (37.5)
Not working	179 (24.9)	131 (28.4)
Studying		
Full-time	313 (43.5)	212 (45.9)
Part-time	362 (50.3)	243 (52.6)
Not studying	45 (6.3)	7 (1.5)
Relationship status		
Single	305 (42.4)	186 (40.3)
De facto	192 (26.7)	118 (25.5)
Married	223 (31.0)	158 (34.2)
Living in Australia	689 (95.7)	449 (97.2)
Current sleep problems	259 (36.0)	189 (40.9)
History of mental illness	221 (30.7)	160 (34.6)
Current physical problems	94 (13.1)	73 (15.8)

*NB. De facto relationships is used in Australia to refer to a couple who lives together but are not married*.

### Materials and procedures

A questionnaire package was developed and delivered online using Qualtrics software (https://www.qualtrics.com/au/), Version 15. Participants were given a direct link to the survey. Order of items in the 170-item pool was randomized prior to participant administration. A single item measuring mindless responding was included: “Some participants don't read questionnaires carefully, please select ‘agree’ for this question.” Ninety-two percent of participants selected the correct response for this item. The 62 incorrect responses were excluded leaving a final sample of 720 participants for item reduction analyses [adequate for an EFA of 170 items Tabachnick and Fidell, 2013]. The university's internal ethics review board approved study procedures.

## Study 1: Results

### Exploratory factor analysis

Preliminary analyses showed the data were amenable to factor analysis. Inspection of the correlation matrix at the item generation stage showed numerous correlations >0.3 with sampling adequacy meeting criteria (Kaiser-Meyer-Olkin = 0.94). Bartlett's test of sphericity indicated that the observed correlation matrix deviated significantly from the identity matrix [$\chi_{\left(14,\;365\right)}^{2}$ = 84692.54, *p* < 0.001].

The enforced orthogonal three-factor solution (explaining 30% of the total variance) was robust against Costello and Osborne's [35] criteria. Inspection of item content and factor loadings in the forced 3-factor solution generated three robust factors with item content of depressed mood, morningness, and sleep quality.

Data-driven approaches to factor extraction generated a wide range of number of factors to extract. Inspection of the scree plot indicated five factors, the minimum average partial test indicated 26 factors, parallel analysis indicated 26 factors, and Kaiser-Guttman's criteria indicated 35 components with eigenvalues greater than 1. None of the empirically derived solutions were robust against the criteria of Costello and Osborne (2005). The 35-factor solution had five stable factors, the 26-factor solutions had four stable factors and the five-factor solution had three stable factors all explored for item content. All factor solutions indicated factor content of: depressed mood, morningness, and sleep quality; the fourth factor in the 26- and 35-factor solution included item content of value and beliefs about sleep, and the 35-factor model had a fifth stable factor with items pertaining to hypomania.

### Item reduction

After initial item removal using the above criteria, 36 items remained on a provisional depressed mood scale, 24 items on a provisional sleep quality scale, and 38 items on a provisional morningness scale. Applying the principles outlined above to select the final items, the scales could be unambiguously named Depressed Mood, Morningness, and Good Sleep on the basis of their item content.

The 15-item Sleep, Circadian Rhythm and Mood (SCRAM) questionnaire had three 5-item scales (one reverse-scored item per scale) (Appendix). As shown in Table 2, these items had moderate-large loadings on their respective factors, and the resultant scales had adequate internal reliabilities, and interpretable intercorrelations in this sample (Table 3).

**Table 2:** Items and Factor Loadings for Morningness, Good Sleep, and Depressed Mood Scales.

**Scale**		**Item**	**Factor Loading**
Morningness	M1	People talk about “morning” and “evening” people, I'm a morning person	0.86
	M2	I work most efficiently before midday	0.75
	M3	Waking up at 7am or earlier works really well for my natural body clock	0.70
	M4	I get very tired by 11pm	0.54
	M5	I wake up at least 2 h later on a day off compared to a work day^*^	−0.42
Good Sleep	GS1	I get the amount of sleep that I need	0.79
	GS2	I sleep soundly through the night	0.68
	GS3	I wake up feeling refreshed, like I've had enough sleep	0.66
	GS4	If I slept better at night my life would be drastically different^*^	−0.54
	GS5	I fall asleep within 30 minutes of trying to sleep	0.49
Depressed Mood	DM1	Everything is going from bad to worse	0.76
	DM2	All I want to do is cry	0.72
	DM3	I have lost interest in things that I used to enjoy	0.65
	DM4	I can't let things go, I find I ruminate a lot	0.50
	DM5	I can laugh and see the funny side of things^*^	−0.44

* *Reverse-scored item*.

**Table 3:** Correlations, Descriptive Statistics and Cronbach's Alpha for the Morningness, Good Sleep and Mood Scales.

**Scale**	**Morningness**	**Good Sleep**	**Depressed Mood**
Morningness	–	0.33^***^	−17^***^
Good Sleep		–	−0.45^***^
Depressed Mood			–
*Mean*	18.05	18.11	11.78
*SD*	5.82	5.51	4.29
Skew	−0.06	−0.25	0.59
Kurtosis	−0.73	−0.69	0.06
Cronbach's α	0.79	0.81	0.77

*N = 720, relevant items reverse scored prior to reliability analysis, theoretical range on each scale is 5 – 30*,

*** *p < 0.001*.

**Figure 1:**
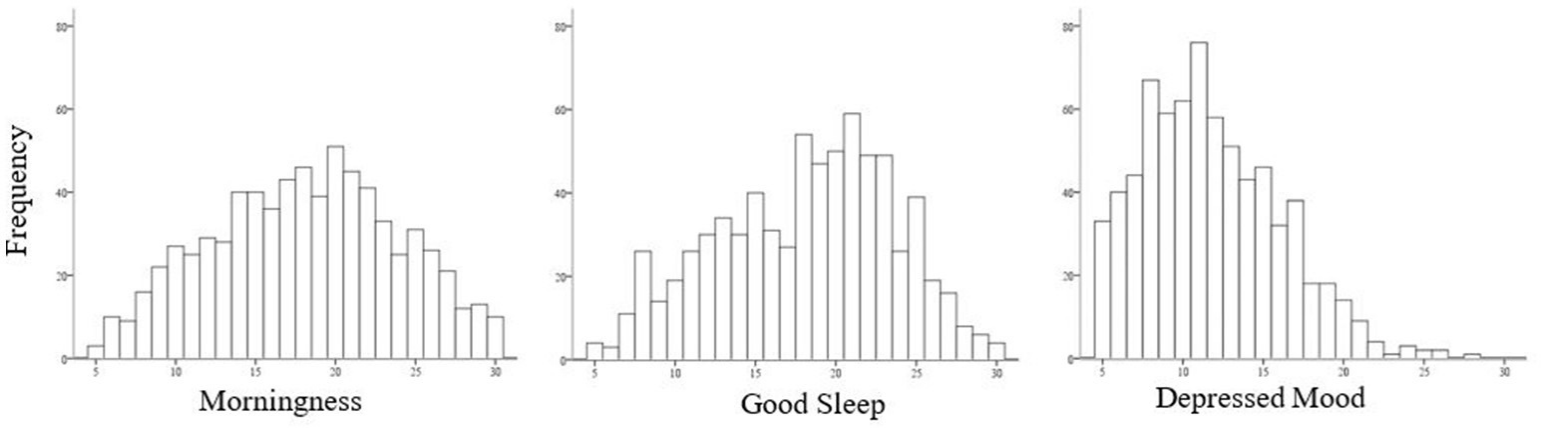
Distribution of scores on factors of Morningness, Good Sleep and Depressed Mood.

Figure 1 shows that scores on the SCRAM Morningness and Good Sleep scales were relatively normally distributed, while a non-significant positive skew was observed for scores on the Depressed Mood scale.

### External correlates

Women scored significantly higher on scores of Morningness than men [*t*_(717)_ = 2.77, *p* = 0.005, Hedge's *g* = 0.27]; and a non-significant trend was observed for women reporting lower levels of Good Sleep [*t*_(717)_ = 1.78, *p* = 0.075]. Scores on Depressed Mood did not differ by gender [*t*_(717)_ = 0.053, *p* = 0.96]. Age was associated positively with Morningness [*r*_(719)_ = 0.21, *p* < 0.001], and negatively with Depressed Mood [*r*_(719)_ = −0.13, *p* = 0.001].

### Preliminary external validation of the SCRAM factors

Independent samples *t-*tests were performed to investigate differences in Morningness, Good Sleep, and Depressed Mood in dichotomous self-reported health outcomes for mental illnesses (31% of the sample), reported physical complaints (13%) and sleep problems (36%) (see Table 4).

**Table 4:** Differences in Mean Good Sleep, Depressed Mood and Morningness Scores Across Self-Reported Mental Illness Status, Physical Complaints, and Sleep Problems.

	**Mental Illness (31%)**	**No Mental Illness (69%)**	***df***	***t***	**Hedges' *g***
Morningness	17.24 (6.03)	18.41 (5.70)	719	2.51^**^	0.20
Good Sleep	16.22 (5.69)	18.94 (5.21)	389.60	6.07^***^	0.51
Depressed Mood	12.95 (4.44)	11.26 (4.12)	719	4.97^***^	0.40
	**Physical Complaints (13%)**	**No Physical Complaints (87%)**			
Morningness	16.89 (5.59)	18.22 (5.84)	719	2.07^*^	0.23
Good Sleep	15.64 (6.17)	18.48 (5.31)	114.58	4.23^***^	0.52
Depressed Mood	12.22 (4.47)	11.71 (4.26)	719	1.08	0.12
	**Problems Sleeping (36%)**	**No Problems Sleeping (64%)**			
Morningness	16.98 (6.14)	18.66 (5.55)	493.47	3.64^***^	0.29
Good Sleep	13.90 (4.67)	20.48 (4.42)	719	18.82^***^	1.46
Depressed Mood	13.20 (4.55)	10.98 (3.92)	473.81	6.61^***^	0.53

*Standard deviations presented in parentheses, adjusted degrees of freedom were used when the homogeneity of variance assumptions was violated*,

* *p < 0.05*,

** *p < 0.01*,

*** *p < 0.001*.

Self-reporting a diagnosed mental illness was associated with lower scores on Morningness [*t*_(718)_ = 2.55, *p* = *0*.011, Hedge's *g* = 0.21] and Good Sleep [*t*_(390.22)_ = 6.07, *p* < 0.001, Hedge's *g* = 0.51], and higher scores on Depressed Mood [*t*_(718)_ = 4.97, *p* < 0.001, Hedge's *g* = 0.40]. Self-reported presence of physical complaints was associated with lower scores on Good Sleep [*t*_(114.65)_ = 4.23, *p* < 0.001, Hedge's *g* = 0.52] and Morningness [*t*_(718)_ = 2.10, *p* = 0.036, Hedge's *g* = 0.23], but was unrelated to Depressed Mood [*t*_(718)_ = 1.07, *p* = 0.28]. Finally, self-reported problems sleeping were associated with lower scores on Good Sleep [*t*_(718)_ = 18.84, *p* < 0.001, Hedge's *g* = 1.46] and Morningness [*t*_(491.89)_ = 3.57 *p* < 0.001, Hedge's *g* = 0.29] and higher scores on Depressed Mood [*t*_(470.83)_ = 6.62, *p* < 0.001, Hedge's *g* = 0.54].

## Study 2: Method

### Participants, materials, and procedure

A predominantly student population was recruited with 498 complete survey responses (95% completion rate). A further 36 participants incorrectly answered the validity question (see Study 1 Method) and were deleted from further analyses. Administration of the questionnaire was identical to Study 1 with participants answering demographic questions and then the draft item pool. From these items the 15 items selected in Study 1 for the SCRAM questionnaire were screened for univariate outliers with 32 participants deleted across the scales to leave a final sample of 430 for the CFA (see Table 1 for sample characteristics).

### Data analyses

Using Mplus version 7.11 (Muthén and Muthén, 2013), a CFA with a maximum likelihood estimation was used to investigate the three-factor model found in Study 1. Multiple methods were used to assess model fit (Hu and Bentler, 1999) including: the chi square test of model fit (divided by the degrees of freedom given the sensitivity to sample size), root mean square error of approximation (RMSEA), standardized root mean square residual (SRMR), comparative goodness-of-fit index (CFI), and Tucker-Lewis index (TLI). The chi square statistic divided by the degrees of freedom should be less than 3 for an acceptable fit (Schermelleh-Engel et al., 2003), RMSEA scores less than 0.05 reflect a good fit, scores less than 0.08 an adequate model fit; and if the upper limit of the 90% confidence interval is 0.08 this adds additional support of model adequacy (Browne and Cudeck, 1992). SRMR scores should be below 0.08 (Hu and Bentler, 1999; Brown, 2015). CFI and TLI values should preferably be above 0.95, however indices in the range of 0.90–0.95 are acceptable (Hu and Bentler, 1999).

## Study 2: Results

**Figure 2:**
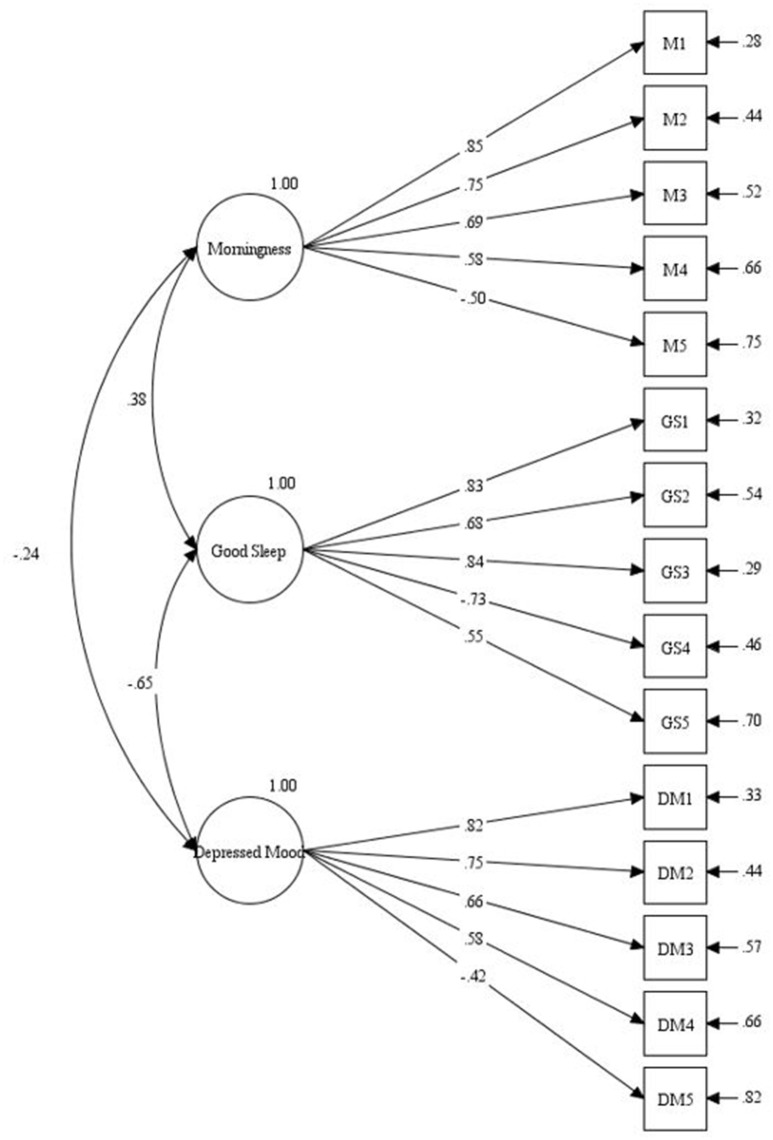
Multiple measurements model of the three factor model.

The results of the CFA are displayed in Figure 2.

The analyses indicated the following fit indices: χ^2^/*df* = 2.93, CFI = 0.93, TLI = 0.92, RMSEA = 0.067 (90% CI [0.058,0.077]), and SRMR = 0.052. Given the reference criteria, model fit was acceptable across all fit indices. As displayed in Figure 2, the resultant model supported the latent structure of the SCRAM as scored. In each case, the five items of the SCRAM scale loaded significantly on a single latent variable. The Morningness scale had a moderate positive relationship to Good Sleep (*r* = 0.38) and a weak negative association with Depression (*r* = −0.24); Good Sleep and Depression had a strong negative relationship (*r* = −0.65).

## General discussion

The aim of this project was to develop the first self-report instrument to reliably measure individual differences in sleep quality, circadian phase, and mood. Three distinct but correlated latent factors of Depressed Mood, Good Sleep and Morningness were identified using exploratory factor analyses. Data-driven factor structures supported the theoretically-based three-factor solution, and systematic item reduction led to the 15-item SCRAM questionnaire—a face-valid instrument maximally separating the measurement of sleep quality, circadian phase and mood. The three-factor latent structure of the SCRAM was confirmed in a second large student sample.

### Relationship between morningness, good sleep and depressed mood scales

Relationships amongst the three SCRAM scales were found to be congruent with existing literature. First, Study 1 and Study 2 data showed that Depressed Mood was correlated with lower levels of Good Sleep. Sleep disturbances affect mood (Pilcher and Huffcutt, 1996), and mood problems have been associated with lowered sleep quality through increased rumination and subsequent arousal at night (Schmidt et al., 2011). The negative correlation between Morningness and Depressed Mood aligns with data suggesting that evening-type individuals are more likely to experience depression, and report more severe depressive symptomatology (Abe et al., 2011; Chan et al., 2014). Morningness and Good Sleep had a moderate positive correlation, consistent with evidence that eveningness is associated with higher levels of insomnia complaints (Wittmann et al., 2006; Chan et al., 2014; Barclay et al., 2016). Consistent with some previous research (e.g., Roenneberg et al., 2003), Morningness on the SCRAM questionnaire was positively associated with older age and female gender (cf. Merikanto et al., 2012, for evidence that men may have higher levels of morningness relative to women).

In a preliminary assessment of convergent and divergent validity (Study 1), the SCRAM scales showed interpretable relationships to self-reported medical, sleep and psychiatric diagnoses. Previous or current mental illness were associated with lower scores on Morningness and Good Sleep scales, and higher scores on the Depressed Mood scale, consistent with evidence for sleep and circadian rhythm disturbances in a range of mental health disorders, (e.g., Harvey et al., 2011; Gershon et al., 2012, 2015; Pritchett et al., 2012; Soehner et al., 2016; Wong et al., 2016). Self-report of mental illness was associated with higher levels of Depressed Mood; aligning with previous data showing mood complaints to be common in mood and anxiety disorders, and schizophrenia (Levinson et al., 1999; Ohayon and Roth, 2003; Taylor et al., 2005; Pritchett et al., 2012). Finally, consistent with links between physical illnesses and biological rhythm function (e.g., Katon, 2003; Ancoli-Israel et al., 2006; Musiek et al., 2015; Grimaldi et al., 2016; Haus et al., 2016), self-reported physical illness was associated lower scores on the Good Sleep and Morningness scales.

The potential clinical significance of distinguishing between sleep quality, circadian phase, and mood is underscored by the SCRAM scores of participants who self-reported sleeping problems in Study 1. Self-reported sleep problems, not surprisingly, were associated with lower scores on the SCRAM Good Sleep scale but were also associated with higher scores on the SCRAM Depressed Mood scale. This is consistent with sleep disturbance being part of the diagnostic constellation of mood disorders in the DSM-5 (American Psychiatric Association, 2013) and higher negative mood levels observed following sleep disturbance in healthy individuals (Tempesta et al., 2015; Raniti et al., 2016). Self-reported sleep problems were also associated with lower scores on the SCRAM Morningness scale, consistent with an association between morningness and better sleep quality (e.g., Wittmann et al., 2006). The significance of this is that when a patient presents with sleep complaints mood problems and circadian phase alignment are critical in both the remittance of sleep problems and maintenance of sleep quality (Harvey et al., 2005; Carney et al., 2009; Crowe et al., 2016). Sleep quality may be effectively treated but if the patient continues to have a delayed sleep cycle or depressed mood these may serve as independent risk factors for future problems with sleep quality, circadian phase, and/or mood.

### The SCRAM questionnaire as a clinical and research tool

With additional validation (see below) the SCRAM questionnaire could improve clinical assessment of symptomatology across the three domains. By distinguishing these three strongly interrelated processes, the SCRAM questionnaire may also be a useful research tool. Malhi and Kuiper (2013) highlight that the substrates of sleep quality, circadian phase, and mood are likely to have different configurations and relationships across psychopathologies. The well-recognized biobehavioral interplay between sleep, circadian function and mood may speak to an under-appreciated superordinate construct or biobehavioral process that could be measured by the SCRAM questionnaire: Murray and colleagues have proposed the Circadian Reward Rhythm as a fundamental motivational process with circadian, sleep and mood manifestations (Murray et al., 2009; see also Alloy et al., 2017).

## Limitations

A number of limitations should be noted. The samples of both studies were predominantly women (82 and 79% for Study 1 and 2 respectively) and students (94 and 98%), so findings may not generalize to the general population. Test-retest reliability was not investigated here, and the SCRAM's sensitivity to change in clinical settings is unknown.

## Conclusions

The present project is a necessary first step in an ongoing program of research. Here, we have developed a psychometrically sound, face-valid brief measure that separates three intrinsically correlated processes for measurement/assessment purposes—the SCRAM questionnaire. To confirm the expected clinical utility of the instrument, more research is required. A critical next phase is to develop SCRAM profiles (pre- and post-treatment) for clinical samples with primary presenting problems in the sleep, circadian phase or mood domains. Subsequently our core prediction—that use of such profiles to drive treatment decisions is superior to current practice based on single-construct instruments—can be tested. With further investigation, the SCRAM questionnaire holds promise as a tool for assessing clinically-significant patterns of disturbance across these three processes.

## Ethics statement

This study was given ethics approval by the Swinburne Human Research Ethics Committee. Participants completed an online survey with completion of the survey implying consent, as detailed in the information statement at the beginning of the survey.

## Author contributions

JB had the original idea and contributed to the study design, collected the data, undertook the statistical analyses, and wrote the first draft of the manuscript. GM and BB contributed to the study design and drafting of the manuscript. All authors contributed to and have approved the final manuscript.

### Conflict of interest statement

The authors declare that the research was conducted in the absence of any commercial or financial relationships that could be construed as a potential conflict of interest.

## Appendix

### SCRAM questionnaire

#### Instructions

**Table A1:** The following questions ask about your sleep, mood and timing of daily activities. Pick the answer which best describes you **over the past 2 weeks**.

		**Strongly Disagree**	**Disagree**	**Somewhat Disagree**	**Somewhat Agree**	**Agree**	**Strongly Agree**
1	I sleep soundly through the night						
2	People talk about “morning” and “evening” people, I'm a morning person						
3	I have lost interest in things that I used to enjoy						
4	I get very tired by 11 pm						
5	I can laugh and see the funny side of things						
6	I wake up feeling refreshed, like I've had enough sleep						
7	All I want to do is cry						
8	I get the amount of sleep that I need						
9	Everything is going from bad to worse						
10	I wake up at least 2 hours later on a day off compared to a work day						
11	I work most efficiently before midday						
12	If I slept better at night my life would be drastically different						
13	I can't let things go, I find I ruminate a lot						
14	Waking up at 7am or earlier works really well for my natural body clock						
15	I fall asleep within 30 minutes of trying to sleep						

**Scoring**

Items 5, 10, and 12 are reverse scored.

*Scores of Good Sleep, Morningness, and Depressed Mood are calculated by summing the scores for the relevant items following reverse scoring*.

*Good Sleep items: 1, 6, 8, 12 (R), 15*.

*Morningness items: 2, 4, 10 (R), 11, 14*.

Depressed Mood items: 3, 5 (R), 7, 9, 13
