# Supplementary material for: Development of a Measure of Sleep, Circadian Rhythms, and Mood: The SCRAM Questionnaire
Source: Front Psychol. 2017 Dec 1;8:2105. doi: 10.3389/fpsyg.2017.02105 (PMC5717824; doi:10.3389/fpsyg.2017.02105)
Supplement: Supplementary file 1 [file Table1.DOCX]

Supplementary Material

List of Preliminary Item Pool

1 On a day off, I struggle to sleep-in to a later time

2 If I had a task/event where I had to be at my physical, psychological and mental best I would want it to be between about 1-5pm

3 I feel rested when I wake up in the morning

4 To help my sleep, I am careful about when I drink coffee

5 I feel most energised and happy in the morning

6 I feel like the smallest disturbance will wake me during the night

7 I normally eat the same amount of meals at roughly the same time each day

8 I get very tired by 11pm

9 I feel drowsy after awakening, even after a good night’s sleep

10 Even if things are bad now I know they’re going to be better in the future

11 People know not to talk to me first thing in the morning because I'm so irritable

12 Even when good things happen I feel like nothing could make me happy

13 I usually need medicine to fall asleep at night

14 I can’t predict whether I’ll have a good or bad night’s sleep

15 Bad dreams have caused me trouble sleeping

16 I remember more of my dreams than I used to

17 I’m so excited about everything, I feel like my thoughts are racing

18 Life is meaningless and I can’t see the point in anything

19 I feel afraid, nervous or scared most of the time

20 It’s been difficult for me to go to work and study

21 When sitting an exam, my peak performance time would be in the evening, after about 5pm

22 I feel most energised and happy in the evening

23 Everything that I do takes a lot of effort

24 I feel relaxed when I go to bed at night

25 I don’t feel refreshed in the morning even if I sleep well

26 I would consider myself an evening person

27 I feel most energised and happy in the afternoon

28 Using medication, alcohol or other drugs helps me get to sleep at night

29 I wake up throughout the night for no particular reason

30 I feel like I’m getting too much sleep

31 My sleep impacts on my ability to work and study during the day

32 Most days I have a nap that lasts at least 30 minutes

33 I care about my sleep

34 If I had a task/event where I had to be at my physical, psychological and mental best I would want it to be after about 5pm

35 I spend at least one and a half hours outdoors on a workday

36 My partner tells me that I disturb their sleep by snoring or other sounds and/or movements

37 When it’s my usual bedtime I can’t overcome sleepiness

38 I have nightmares and remember what they are about

39 I love sleeping

40 I’m satisfied with my current sleep

41 People have been saying I’m louder and more disruptive than normal

42 It takes a lot to get me angry or upset, little things don’t bother me

43 I toss and turn in bed a lot

44 I can work just as easily during the day as at night

45 I would like to be more alert in the evening

46 I’m really satisfied with my sleep

47 I find it easy to find the motivation to get things done

48 Everything is going from bad to worse

49 I spend time in bed thinking about exciting things that have happened or are to come

50 I have felt much more self-confident than usual

51 I get the amount of sleep that I need

52 I worry about my sleep a lot

53 I have felt really enthusiastic, energetic and joyful

54 I can’t let things go, I find I ruminate a lot

55 I don’t think I’m a morning person or an evening person, I’m somewhere in the middle

56 I feel like I need a nap in the afternoon

57 I use my bed for lots of things: studying, eating, watching TV etc.

58 I can’t stop thinking about whether people like me

59 I’d be more productive if I could start work later and finish later

60 The only time I use my bed is to sleep

61 I’m really satisfied with my life, things are really good

62 As soon as my head hits the pillow I’m asleep

63 I often fall asleep unintentionally or fight to stay awake during the day

64 I’d much rather start work earlier and finish earlier

65 All I want to do is cry

66 Compared to normal, I can’t make simple decisions or concentrate

67 I’ve had restless or ‘crawling’ feelings in my legs at night that goes away if I move them

68 Compared to most people my age I have an early bedtime

69 I feel like I’m a burden to everyone around me

70 I feel like I am to blame for everything

71 If I’ve slept well it doesn’t take me long to wake up

72 On holidays my sleep always drifts to later times

73 I would like to wake up earlier

74 I feel like I can’t do anything without help

75 A 9-5 work-day suits me well

76 I normally leave the house at the same time each morning

77 I wake up feeling refreshed, like I’ve had enough sleep

78 I get enough sleep on weekdays, so I don’t need to catch up on weekends

79 I look forward to seeing my friends and relatives who I care about

80 When I wake up on a day off I’m really hungry

81 I am more irritable and ‘snappy’ than usual

82 I’d be lucky to spend more than 30 minutes outside most days

83 If I had a task/event where I had to be at my physical, psychological and mental best I would want it to be before about 2pm

84 I sleep longer on weekends than workdays

85 I’ve noticed it takes me longer to fall asleep

86 Waking up at 7am or earlier works really well for my natural body clock

87 I think sleep is really important to my well-being

88 I feel so agitated and worried I can barely sit still

89 I have quite a regular schedule with work and social activities

90 When I have a day off, I would get less than 4 hours outside in daylight

91 No matter how much I eat, I’m still hungry

92 I’d describe my mood as calm and content

93 It takes me a long time to get to sleep

94 I spend a lot of time in bed at night worrying about bad things

95 I have felt so excited I can barely sit still

96 I feel so alone but I’m also pushing away people I love

97 My bedtime (including weekends) does not change by more than 2 hours

98 I have lost interest in things that I used to enjoy

99 Having regular times for daily activities helps me to sleep better

100 I don’t want to be near anyone, even people who I care deeply about

101 My alarm clock is more of a safety net, I rarely need it

102 Once I fall asleep, I don’t wake up throughout the night

103 When sitting an exam, my peak performance time would be in the afternoon, between about 1-5pm

104 I’m more likely to get emotional either crying or becoming irritable in the evening

105 When sitting an exam, my peak performance time would be in the morning, before about 1pm

106 I always get more sleep on the weekend

107 I feel really relaxed when I go to bed at night

108 I’ve been thinking I’m more important, talented and special than other people

109 I feel confident and secure in my relationships

110 I have found it really difficult to concentrate because I’m so sad

111 I fall asleep within 30 minutes of trying to sleep

112 I would struggle to stay awake until 1am

113 I have a lot of variation in what time I go to bed and wake up each day

114 I am worried that I will lose control over my sleep

115 My meal times vary dramatically, there are no set times

116 I’m relaxed and easy-going

117 I wake up at least two hours later on a day off compared to a work day

118 People see me as optimistic and cheery

119 I’ve been told I snore a lot

120 My sleep benefits from waking up at about the same time each day

121 If I had to stay up all night I could function the next day better than most

122 I think my sleep quality is awful

123 I could easily fall into a pattern of later bedtimes if I let myself

124 I don’t understand people who ‘snooze’, when I wake up I’m awake

125 I’ve been thinking about sex more frequently than normal

126 I don’t think much about sleep

127 I have no appetite at the moment

128 Sleeping badly one night means I’ll sleep badly all week

129 If I do exercise I prefer it to be first thing in the morning

130 If I have work and study to catch up on, I’d rather wake up early than work late

131 Sleep is really low on my priority list

132 I’ve been patient with people whose words or actions are irritating

133 I can laugh and see the funny side of things

134 I feel lonely, down-hearted and blue

135 Exercising is part of my routine

136 I am so tired that I struggle to stay awake for meals, driving and social occasions

137 When I sleep badly I need to catch up the next day by napping or sleeping longer the next night

138 I’m particularly sensitive to any rejection or criticism

139 I feel like my future is hopeless and it will just get worse

140 I feel like I am in control of my sleep

141 I get really upset over little things

142 External factors (such as light, noise or temperature) make it really difficult for me to fall asleep

143 People say I’m easily distracted and that it’s difficult to follow my train of thought

144 I always need an alarm clock to wake up in the morning

145 I don’t enjoy eating in the morning, if I do it’s out of habit

146 If I wake at 9am I would feel sluggish, slowly warming up throughout the day

147 I feel alert and most energetic in the morning, over the day my energy runs out

148 I have felt that others would be better off if I were dead

149 I think I’m most alert first thing in the morning

150 I can’t manage the negative consequences of poor sleep

151 I feel weighed down, like I’m doing everything in slow-motion

152 People talk about ‘morning’ and ‘evening’ people, I’m a morning person

153 I sleep soundly through the night

154 When I go to bed at night I feel really calm

155 When I have a day off the following day I always stay up later

156 I can’t schedule meetings or classes in the morning as I worry I’ll sleep through them

157 I feel like I have really disturbed sleep at night

158 If I nap during the day I sleep badly that night

159 Sleep is a really important part of my life

160 I have a really depressed mood

161 I hate myself

162 7 hours sleep is more than enough for me

163 I stay up a lot later when I have no commitments the following day

164 I have been feeling unusually good, cheerful, or happy

165 I’m not willing to change my sleep during the week if it means I can’t stay up as late on weekends

166 Some participants don't read questionnaires carefully, please select 'agree' for this question*

167 I have to set multiple alarms each morning

168 I feel more irritable, hostile and angry than normal

169 If I slept better at night my life would be drastically different

170 I work most efficiently before midday

171 When I’ve slept badly, I can hardly function the next day

*Please note this item was used as a validity question to measure mindless responding in participants
